# Protein Hydrolysis as a Way to Valorise Squid-Processing Byproducts: Obtaining and Identification of ACE, DPP-IV and PEP Inhibitory Peptides

**DOI:** 10.3390/md22040156

**Published:** 2024-03-28

**Authors:** Hajer Bougatef, Assaad Sila, Ali Bougatef, Oscar Martínez-Alvarez

**Affiliations:** 1 Laboratory for the Improvement of Plants and Valorization of Agroresources, National School of Engineering of Sfax (ENIS), University of Sfax, Sfax 3038, Tunisia; hajer.bougatf93@gmail.com (H.B.); saadsila@gmail.com (A.S.); ali.bougatef@isbs.usf.tn (A.B.); 2 Department of Life Sciences, Faculty of Sciences of Gafsa, University of Gafsa, Gafsa 2100, Tunisia; 3 High Institute of Biotechnology of Sfax, University of Sfax, Sfax 3038, Tunisia; 4 Institute of Food Science, Technology and Nutrition (ICTAN, CSIC), 6 José Antonio Novais St, 28040 Madrid, Spain

**Keywords:** squid byproducts, ACE, DPP-IV, Prolyl Oligopeptidase, bioactive peptides, protein hydrolysates, seafood upgrading, functional ingredients

## Abstract

The industrial processing of Argentine shortfin squid to obtain rings generates a significant amount of protein-rich waste, including the skin, which is rich in collagen and attached myofibrillar proteins. This waste is generally discarded. In this study, skin was used as a source of proteins that were hydrolysed using Trypsin, Esperase® or Alcalase®, which released peptides with antioxidant potential and, in particular, antihypertensive (ACE inhibition), hypoglycemic (DPP-IV inhibition) and/or nootropic (PEP inhibition) potential. Among the three enzymes tested, Esperase® and Alcalase produced hydrolysates with potent ACE-, DPP-IV- and PEP-inhibiting properties. These hydrolysates underwent chromatography fractionation, and the composition of the most bioactive fractions was analysed using HPLC-MS-MS. The fractions with the highest bioactivity exhibited very low IC50 values (16 and 66 µg/mL for ACE inhibition, 97 µg/mL for DPP-IV inhibition and 55 µg/mL for PEP inhibition) and were mainly derived from the hydrolysate obtained using Esperase®. The presence of Leu at the C-terminal appeared to be crucial for the ACE inhibitory activity of these fractions. The DPP-IV inhibitory activity of peptides seemed to be determined by the presence of Pro or Ala in the second position from the N-terminus, and Gly and/or Pro in the last C-terminal positions. Similarly, the presence of Pro in the peptides present in the best PEP inhibitory fraction seemed to be important in the inhibitory effect. These results demonstrate that the skin of the Argentine shortfin squid is a valuable source of bioactive peptides, suitable for incorporation into human nutrition as nutraceuticals and food supplements.

## 1. Introduction

Marine fish and invertebrates comprise approximately 50% of the world’s biodiversity and offer a plethora of bioactive and functional compounds, including proteins, peptides, and amino acids. In addition to being a primary source of high-quality protein, marine species are also utilized as a source of bioactive peptides [1].

Cephalopods are of particular interest due to their nutritional value for human health and commercial significance. The global market for cephalopods has grown in recent years. According to the Food and Agriculture Organization [2], cephalopods account for 11% of the world trade in fishery products. Squid species from the Ommastephidae family, such as the Pacific flying squid and jumbo squid, are the most significant group of cephalopods in the neritic and oceanic zones due to their high commercial fishing volume [3]. In recent decades, they have been gaining economic importance, as evidenced by their rapid increase in global fishing yields.

The squid processing industry generates substantial waste, posing environmental challenges. It is crucial to address this issue within a circular economy framework [4]. Scientists emphasize the importance of minimizing fishing-related waste for environmental conservation. Integrating these byproducts efficiently can help reduce the large volume of waste discarded by the fishing industry.

Marine byproducts, including squid skins, are a rich source of bioactive peptides that can be released from native proteins through hydrolysis [5,6]. These peptides have various beneficial characteristics, including antioxidant properties, the potential to regulate calcium, the potential to provide antihypertensive and antidiabetic effects and neuroprotective benefits [7]. Collagen, a vital protein found in squid skin, has the potential to be a valuable source of bioactive peptides due to its unique structure and properties. The food, pharmaceutical and cosmetic industries have all utilized this polymer because of its gelling and surface properties, excellent biocompatibility, and numerous biological, biochemical and biomedical functions [8]. The preferred method for releasing bioactive peptides from collagen is through controlled enzymatic hydrolysis [9]. 

*Illex argentinus*, commonly known as the Argentine shortfin squid, is the most abundant squid species in the Atlantic Ocean [10], which is representative of a productive fishery in terms of capacity and economic yield. Over the past ten years, this species has been commercially exploited in many regions of the Northeast Atlantic Ocean and Mediterranean sea. As with any other marine species, industrial cleaning and processing produce significant amounts of byproducts that are rich in various nutrients, such as proteins, lipids, minerals, vitamins, enzymes and biopolymers. This project aims to maximize the value of a significant industrial byproduct, the Argentine shortfin squid, by exploring its potential as a source of functional ingredients. These include antioxidant, anti-hypertensive (Angiotensin Converting Enzyme I inhibitors), hypoglycemic (Dipeptidyl Peptidase IV inhibitors) and nootropic peptides (Prolyl Endopeptidase inhibitors) through controlled protein hydrolysis. Additionally, the study aimed to identify the peptides in the hydrolysates that may be responsible for their bioactive properties.

## 2. Results and Discussion

### 2.1. Proximate Composition of the Squid Skin and Amino Acid Composition

The skin had a high moisture content of 84.18 ± 0.23%, and a low ash content of 0.85 ± 0.17%. The total carbohydrates content was approximately 2.71%. The protein content was approximately 12.25%, which is consistent with measurements of protein in the skin of the Pacific flying squid [11]. 

A database of 1742 protein sequences or fragments present in the raw material was obtained through sequencing (data not shown). The most abundant sequences corresponded to actin or fragments of this protein, indicating that the byproducts used as raw materials were composed of skin and muscle attached. Other proteins, including tropomyosin, histones, myosin, paramyosin and collagen, were also present in intact or fragmented form.

Table 1 displays the amino acid composition of the raw material, with the most abundant amino acids being Gly (215 ‰), Glx (98 ‰) and Asx (87 ‰). The raw material’s high concentration of Glu and Asp is attributed to the presence of myofibrillar proteins, which are abundant in the mantle, as described in various cephalopod species [12]. Additionally, the presence of these amino acids in protein hydrolysates is possibly of interest due to their potential antioxidant properties [13]. The abundance of Ala is mainly due to the presence of collagen. The lower percentage of main residues in squid skin gelatine may be due to the presence of other proteins in the raw material. The Gly content was high but lower than that found in purified skin gelatine from *Dosidicus gigas* (33.6%), as reported by Gómez-Guillen et al. [14]. Additionally, the Hyp and Pro contents were lower than those described in pure squid gelatine (7.8% and 10.7%, respectively). The raw material contained 32.5% essential amino acids and approximately 51% hydrophobic amino acids. The hydrophobic amino acid content of squid protein is of considerable interest due to its association with bioactive properties such as ACE or PEP inhibition. Moreover, the presence of hydrophobic amino acids in hydrolysates has been linked to a high potential for free-radical scavenging, as hydrophobic amino acids enhance the solubility of peptides in non-polar media [15]. However, their presence has not been correlated with good ferric-reducing antioxidant properties [13].

### 2.2. Degree of Hydrolysis (DH) and Molecular Weight Distribution

Three proteases with distinct substrate specificities were used to hydrolyse the protein in squid skin. These enzymes have previously been used to digest proteins from various fishery byproducts [16]. Among these proteases, the hydrolysate obtained using Esperase (hydrolysate E) exhibited a greater ability to hydrolyse squid skin protein, resulting in a DH of 13.25%. This could be attributed to its broad substrate specificity. Vázquez et al. [16] documented that Esperase achieved a higher DH than Alcalase when applied to squid processing byproducts. Alcalase demonstrated a high capacity for hydrolysis, but resulted in a lower DH value of the hydrolysate (hydrolysate A, 9.51%). This finding is consistent with Segura Campos et al. [17], who reported that the use of Alcalase does not result in DH values higher than 20–25%. Esperase has a higher hydrolytic capacity due to factors such as a more flexible substrate binding site [18], different substrate specificity [19] or the use of more suitable hydrolysis conditions. The hydrolysate obtained using Trypsin (hydrolysate T), on the other hand, showed the lowest DH value (5.65%) possibly due to its limited substrate specificity. Trypsin can only cleave on the carboxyl side of lysine or arginine, which are present in moderate amounts in the raw material [20]. However, in their study, Choonpicharn et al. [21] found that Trypsin has a higher hydrolytic capacity than Alcalase when using Nile tilapia skin gelatine as the substrate. Similarly, Shavandi et al. [22] investigated the impact of different proteases on the degree of hydrolysis (DH) using a squid processing byproduct (pen), and found that Pepsin yielded the highest DH compared to Trypsin and Alcalase. These findings indicate that the final DH can be affected by the hydrolysis conditions and protein conformation, which may impede the accessibility of the proteases for the peptide bonds [23].

The degree of hydrolysis and the specificity of cleavage can significantly affect the ACE inhibitory activity of the hydrolysates. This is because both factors determine the length and amino acid composition of the peptides that are released [24]. Table 2 shows the molecular weight distribution of the hydrolysates, indicating that the use of Esperase resulted in the highest concentration of low-molecular-weight peptides, which is consistent with achieving the highest degree of hydrolysis. This hydrolysate was mainly composed of peptides around 720 Da. The other hydrolysates were mainly composed of peptides above 1 kDa, proportional to the degree of hydrolysis. Thus, the major components in the hydrolysate A were peptides around 1.1 kDa, while the hydrolysate T was mainly composed of peptides greater than 2.5 kDa. Peptide molecular weight has been emphasized by various researchers as significant in the bioactive properties of hydrolysates. For instance, Zheng et al. [25] showed that peptides with low molecular weight have increased ACE inhibitory activity. Likewise, Alemán et al. [26] reported significant ACE activity inhibition from peptides derived from hydrolysed squid gelatine with a molecular weight lower than 1.4 kDa. Additionally, Nuchprapha et al. [27] found that the fraction containing peptides with a molecular weight of less than 1 kDa from longan seed proteins concentrated the most potent ACE inhibitory peptides.

### 2.3. Biological Properties of Protein Hydrolysates

#### 2.3.1. Angiotensin-I-Converting Enzyme Inhibitory Activity

ACE is an enzyme that converts angiotensin I into angiotensin II, which increase vasoconstrictor action and consequently blood pressure. Different synthetic ACE inhibitor peptides, such as Captopril or Lisinopril, are commonly used as antihypertensive drugs, but they have some side effects. Although natural peptides have not demonstrated similar effectiveness to these drugs, they are potentially used as mild antihypertensive drugs in the form of healthy ingredients. In this work, all the hydrolysates showed a potent ACE inhibitory capacity, with very low IC50 values that ranged from 40 to 70 µg/mL (Table 2). The hydrolysate A showed the lowest values, followed by the hydrolysates E and T. The comparable ACE inhibition capacity of protein hydrolysates with varying DH and MW profiles suggests that achieving high levels of hydrolysis and an increased release of small peptides are not necessary to attain high levels of inhibition. The size and sequence of peptides are crucial in ACE-inhibiting activity. Low-molecular-weight peptides, specifically those less than approximately 1000 Da, exhibit a better ACE inhibitory activity. The inhibitory activity of the hydrolysate T, composed primarily of peptides with a molecular weight higher than 1 kDa, may be worse due to the difficulty for larger peptides to reach the active site [28]. However, the better inhibitory activity of the hydrolysate A, with an average molecular weight slightly higher than 1.1 kDa, cannot be explained solely by this factor. This statement suggests that hydrolysate A contains peptides with a more effective inhibitory power due to the different sequence, and this would be the result of a different cleavage specificity of this enzyme. Segura-Campos et al. [17] observed that using Alcalase to hydrolyse protein releases peptides with predominantly branched-chain amino acids at the C-terminal end. It is known that the presence of hydrophobic residues at C-terminal positions is significant for the ACE inhibitory activity [29]. The presence of branched-chain aliphatic amino acids in the N-terminus is important for the inhibitory capacity of the peptides [30,31]. This confirms the significance of the protein sequence being hydrolysed and the cleavage specificity of the enzyme being used to release ACE inhibitory peptides.

The IC50 values reported in this study (Table 2) were significantly lower than those reported by Liu et al. [32] for various hydrolysates of squid protein, as well as those reported by He et al. [33] for 48 marine protein hydrolysates (ranging from 0.17 to 501.7 mg/mL). Moreover, the IC50 values were much lower than those reported by Ko et al. [34] for flounder muscle hydrolysates obtained with Pepsin, Trypsin and Papain (with IC50 values of 1.26, 1.47 and 2.14 mg/mL). Compared to the giant squid hydrolysates prepared by Alemán et al. [26], which had IC50 values ranging from 0.34 to 1.6 mg/mL, the hydrolysates of squid skin protein also exhibited greater ACE-inhibiting activity. Furthermore, the IC50 values obtained were lower than those presented by Lin et al. [24] for a squid protein hydrolysate before and after ultrafiltration (IC50 values of 0.96 mg/mL and 0.33 mg/mL, respectively). The values were also lower than those reported by Mosquera et al. [35] for different peptidic fractions from a hydrolysate of a giant squid tunic (IC50 values of 51–93 µg/mL).

#### 2.3.2. DPP-IV Inhibitory Activity

DPP-IV is an enzyme that regulates blood glucose levels by breaking down the incretins GLP-1 and GIP. This results in reduced insulin secretion and increased glycemia [36]. DPP-IV inhibitors such as sitagliptin, alogliptin or saxagliptin block the degradation of GLP-1 and GIP, promoting glycemic control in individuals with type 2 diabetes. However, these drugs usually have side effects, such as increased heart failure or joint pain, so the search for natural and safe DPP-IV-inhibiting peptides that could exert in vivo effects is of interest. Various DPP-IV inhibitory peptides have been obtained by hydrolysing protein from different sources, including marine organisms, plants and milk [36]. In this study, skin-protein hydrolysates showed varying levels of DPP-IV inhibitory potency (Table 2). The hydrolysate E showed the highest inhibitory capacity (IC50 of 673 µg/mL), followed closely by the hydrolysates A and T. It agrees with the finding of Jin et al. [37], which reported a positive correlation between the degree of hydrolysis and the DPP-IV inhibitory capacity. The lowest DPP-IV inhibitory capacity of hydrolysate T differs from the results reported by Jin et al. [37], who found a greater inhibitory capacity in a salmon hydrolysate produced with Trypsin compared to protein hydrolysates produced with alternative enzymes such as Alcalase. The study demonstrated that the use of Trypsin on a hydrolyse protein resulted in a higher DH value. This highlights the importance not only of enzyme selection, but also of the protein used and the DH achieved in the production of potent DPP-IV inhibitory hydrolysates. 

#### 2.3.3. PEP Inhibitory Activity

PEP is a post-proline cleaving enzyme that degrades neuroactive peptides and is involved in neurocognitive diseases. The inhibition of PEP has been considered as a potential treatment for neurodegenerative disorders. In vivo experiments have demonstrated anti-amnesic effects of PEP inhibitors in rats [38]. Most PEP inhibitors described in the literature are compounds with a pyrrolidine or a pyrrolidine ring substituted at the P1 site [39]. In the present study, the protein hydrolysates exhibited significant PEP inhibitory activity, especially E, which showed the lowest IC50 value (38 µg/mL). Compared to various barbell skin gelatine hydrolysates (with IC50 values ranging from 0.91 to 3.79 mg/mL), the hydrolysates showed greater PEP inhibitory activity [40]. The results suggest that the DH may be important in obtaining potent PEP inhibitory peptides. The importance of the DH on the inhibitory activity has also been reported by Taraszkiewicz et al. [38], who found that most PEP inhibitory peptides have a short length and low molecular weight. Lajmi et al. [41] also observed the importance of low-molecular-weight peptides in the PEP inhibitory activity of protein hydrolysates. However, the similar PEP inhibitory activity of hydrolysates A and T suggests that factors other than enzyme type, such as peptide sequence, may also play a role in the inhibition. This observation is consistent with previous studies [41,42]. Martínez-Alvarez et al. [42] observed that the PEP inhibitory capacity of tuna head and viscera hydrolysates was significantly enhanced after gastric digestion due to the release of smaller peptides, while it remained unchanged after further intestinal digestion. This supports the relevance of the presence of specific residues at particular positions in the peptide chain for PEP inhibitory activity.

#### 2.3.4. Antioxidant Activity

The antioxidant activity of protein hydrolysates was evaluated using ABTS and FRAP assays. The ABTS assay measures the ability of a compound to donate protons and electrons, whereas the FRAP assay determines the capacity of an antioxidant to donate electrons. The high content of hydrophobic amino acids, aspartic acid and glutamic acid in the raw material has been associated with a high antioxidant effect [13,43], so it was thought that the protein hydrolysates should have a high antioxidant activity. However, the results showed that all protein hydrolysates possess a moderate ABTS radical scavenging ability, ranging from 10.76 (hydrolysate A) to 18.68 mg Eq ascorbic acid/mL (hydrolysate E) (Table 2). Moreover, the ferric-reducing antioxidant power of all samples was found to be low. These findings suggest that the arrangement of amino acids in the peptide sequence plays a crucial role in determining the antioxidant activity of the hydrolysates. Furthermore, both the ABTS and FRAP assays revealed no correlation between DH and the antioxidant activity of protein hydrolysates. This suggests that the length of the peptides released did not play a role in the antioxidant effect.

### 2.4. Purification and Biological Activities of Fractions

The hydrolysates A and E showed significant ACE, DPP-IV and PEP inhibitory activity. As a result, they were chosen for the identification of bioactive peptides. The hydrolysates were fractionated by chromatography, and the potential bioactivity of each fraction was evaluated (Table 3). All fractions exhibited potential bioactivity, although with varying degrees of intensity. Fractions 9 and 8 from the hydrolysates E and A respectively, showed the most effective ACE-inhibiting activity, with inhibiting values higher than 50% at very low concentrations (25 µg/mL). Notably, the same fraction from the hydrolysate E, along with fraction 2 from the same hydrolysate, exhibited the highest DPP-IV-inhibiting activity (higher than 80%), although the concentration of the tested fraction was higher (0.8 mg/mL). Fraction 9 of the hydrolysate Esperase and fraction 8 of the hydrolysate A showed the highest PEP inhibitory activity, with values exceeding 50% at a concentration of 0.4 mg/mL. Therefore, the fractions demonstrated superior ACE inhibitory activity compared to that of DPP-IV and PEP.

The fractions with the highest bioactivity were further fractionated and analysed (Table 4). It is worth noting that two fractions that showed a strong inhibition of DPP-IV and PEP did not exhibit activity at the initial concentration tested (30 and 60 µg/mL, respectively). The re-fractionation did not significantly increase the ACE-inhibiting activity, indicating that the potent ACE-inhibiting peptides were also present in other fractions. Nonetheless, the IC50 values obtained were very low, particularly for subfraction 9(1) from the hydrolysate E (Table 4). The subfraction 9(6), also derived from the hydrolysate E, showed potent PEP inhibitory activity, which was significantly increased compared to the values obtained from the first fractionation (IC50 value around 55 µg/mL). Re-fractionation was also effective in isolating the primary peptides responsible for the DPP-IV-inhibiting activity in fraction 2 of the hydrolysate E, which significantly increased from the original fractionation (IC50 value around 97 µg/mL).

### 2.5. Identification of the Peptide Composition of the Most Potent ACE-Inhibiting Fractions

The peptide composition of the fractions with the highest inhibitory activity was analysed, and the results are presented in Table 5 and Table 6. Subfraction 8(1) showed a high ACE inhibitory capacity, with IC50 values of 66 µg/mL. This subfraction consisted of 233 peptides, of which about 75% contained hydrophobic residues in any of the last three positions of the C-terminal end. Twenty of these peptides were more abundant and constituted approximately 60% of the total (Table 5). These peptides were composed of between 7 and 21 residues, with a majority of 7 to 12 residues, and ranged in mass from 755 to 2055 Da. About 50% were novel peptides, while others matched with proteins found in other databases. The three most abundant peptides share the same residues in the last positions of the terminal carboxyl end (GL), accompanied by hydroxyproline in the case of the two most abundant (EGRGLP(+15.99)GL and LGRGEP(+15.99)GL). Both peptides, which make up just over 25% of the total, are part of the *Sephia pharaonis* COL2A protein sequence (A0A812EDK2_SEPPH, source Uniprot). Within the 20 most abundant peptides, Leucine was frequently found in the C-terminal position (8 out of 20 peptides, 59% of the total), often accompanied by glycine (G) in C2 or C3 (10 out of 20 peptides, 74% of the total). This agrees with Segura-Campos et al. [17], who indicated that Alcalase tends to release peptides with hydrophobic amino acids at the C-terminal position. Regarding the N-terminal end, there was no clear predominance of any specific residue. In this position, glycine (4) and leucine (3) were the most abundant residues.

Subfraction 9(1) of the hydrolysate E exhibited a high inhibitory capacity of ACE, with a very low IC50 value (16 µg/mL) that was 4 times lower than that of subfraction 8(1) (Table 4). It consisted of 154 peptides, the majority of which were present at low concentrations. The twenty most abundant peptides are listed in Table 6 (molecular mass ranging from 680 to 2798 Da). Peptides SHPDLRLA, LGMP(+15.99)GRL and GNK(+15.99)GPVGPL accounted for just over 25% of the total. The first and second peptides are novel. The third one was sequenced as a novel peptide, but it has also been identified as a fragment of the *Sephia pharaonis* proteins COL5S, ColAa and Col4A (accession numbers A0A812EPU5_SEPPH, A0A159BRC2_SEPPH, A0A812DMC6_SEPPH, respectively, source Uniprot). Among the identified peptides, 75.4% contained at least one hydrophobic residue in C1, C2 and/or C3. For the most abundant peptides (Table 6), the percentage was 95%. As seen in subfraction 8(1), the presence of leucine at these positions was important, especially in C1 and C2 (6 and 3 of 20 peptides, 42% and 19% of the total, respectively).

ACE inhibitory peptides generally have a molecular weight below 1000 Da [28]. Although ACE inhibitory peptides of higher molecular weight have been described, it is likely that the largest peptides in the analysed subfractions were not the major ACE inhibitors. Other than size, the presence of specific residues at the C-terminal positions is crucial for the binding to ACE. Thus, the presence of specific residues in positions C1, C2, C3 and C4 in peptides ranging from 5–10 residues determines the potency of interaction with active ACE sites and thus the inhibitory potency of the peptide [44]. These authors postulated that the presence of Y and C in position C1; H, W, M in C2; I, L, V, M in C3; and W in C4 is common for impotent ACE-inhibiting peptides. However, these residues were not found in the peptides present in the two subfractions. Several authors [39,45,46,47] have suggested that the presence of hydrophobic amino acids (aromatics or branched chain) in positions C1, C2 and/or C3 is important for binding to ACE and exerts competitive inhibition of the enzyme. The majority of the peptides in 8(1) and 9(1) contained hydrophobic residues in these positions, mainly leucine. Wang et al. [48] previously reported the presence of branched-chain residues in the C-terminal position (C1) of ACE inhibitory peptides. Wu et al. [49] reported that peptides with higher hydrophobicity exhibit a greater inhibitory activity. Other studies [39,50,51,52] have also shown that peptides containing leucine have significant ACE inhibitory potential. Olalere et al. [53] analysed the profile of all peptides described as ACE inhibitors in the BIOPEP-UWM virtual database [54], and found that the presence of branched aliphatic amino acids, such as leucine, in the C1 position was highly noticeable in ACE inhibitory peptides. Based on these studies, it appears that peptides in subfractions 8(1) and 9(1) containing leucine in C1 could be the main ACE inhibitors present in this subfraction. Furthermore, the presence of leucine in certain peptides in the N1 position may also be relevant for the ACE-inhibiting mechanism of the peptides [55].

The presence of proline in peptides could also be important for their inhibitory activity. Olalere et al. [53] found that ACE inhibitory peptides of more than 5 residues usually contain proline, particularly in the C-terminal position. It allows the peptide to bind to ACE by interacting with different residues present in active centres, such as His353, His383, His386 or His513. However, proline was found to be scarce in this position in the analysed subfractions. In contrast, proline was found in position C2 in 28.1% of the peptides of 9(1), decreasing to 15.4% in 8(1). Its presence in this position appears to be crucial for the reactivity of the peptides [53]. Glycine was particularly abundant in C3, present in 45.6% of the peptides in 9(1) and to a lesser extent in the peptides of 8(1), with 23.3%. Although its role does not appear to be significant in the interaction of the peptides with ACE, it may play a role in their inhibitory potential. According to Olalere et al. [53], the presence of proline, valine or alanine in C3 is crucial for the inhibitory capacity of peptides greater than 5 residues. However, their presence was insignificant in the peptides of the analysed fractions. Therefore, it is likely that both fractions have ACE inhibitory potential due to the high presence of hydrophobic residues in most peptides, particularly leucine, or the presence of proline in certain C-terminal positions.

### 2.6. Identification of the Peptide Composition of the Most Potent DPP-IV-Inhibiting Fractions

The hydrolysate E showed a strong inhibitory capacity of DPP-IV with an IC50 of 97 µg/mL (Table 7). The majority of the identified peptides corresponded to proteins present in the database, while the presence of novel peptides was minimal. The 18 most-abundant peptides represented just over 96% of the total. They were composed of 9 to 15 residues. This agrees with the review published by Nong and Hsu [36], who indicated that the DPP-IV inhibitory peptides identified prior to publication were composed of 2 to 15 residues. Their molecular mass ranged from 796 to 1728 Da. The most abundant peptide, which represented 21.6% of the total, contained 3 hydroxyprolines (GEP(+15.99)GEQGPP(+15.99)GSP(+15.99)GPR). This peptide is a fragment of the ColAbprotein (A0A159BRA1_SEPPH, from Uniprot) of *Sephia pharaonis*. It is noteworthy that 11 out of 18 peptides (63.6%) had a glycine residue at the N-terminal, which was almost always accompanied by alanine or proline as the second residue (29.2%). Glycine was particularly abundant in position C3 (10 out of 18, 68.6%), followed by proline (hydroxylated or non-hydroxylated) in C2 (6 out of 18, 52.3%) and arginine in the C-terminal position (8 out of 18, 54%). As well, proline was found in the third position from the N-terminal in 7 of the 18 most abundant peptides (20.8% of the total). 

DPP-IV inhibitory peptides can exert their inhibitory effect via different mechanisms depending on their ability to bind to different domains of the enzyme. Nong and Hsu [36] and Mu et al. [56] suggested that the presence of certain residues in the N-terminal position is important for DPP-IV inhibition. Specifically, Gly, Ala, Pro, Ile, Leu, Met, Val or Glu residues interact with the hydrophobic pocket of the active site. Peptides with glycine in this position were particularly relevant (Table 7). The presence of proline or alanine at the second N-terminal position favors DPP-IV inhibition due to the affinity of the enzyme for these ligands [56]. In the fraction, it was observed in about 30% of the peptides, for example in GADGERGPRGNRGDTGAS, GPAGP(+15.99)P(+15.99)GAPG or GPTGP(+15.99)AGPAG (Table 7). According to Nong and Hsu [36], almost all food-derived peptides that are DPP-IV inhibitors act as substrates. Peptides with proline in the third position also inhibit the enzyme because it cannot catalyse the hydrolysis of such bonds [56,57]. Furthermore, the presence of proline, leucine or glycine in positions 1–4 from the C-terminus seems to be related to the inhibitory activity [58]. Almost all peptides in the subfraction contained one or more of these residues in these positions (Table 7), suggesting that they are responsible for the DPP-IV-inhibiting activity.

### 2.7. Identification of the Peptide Composition of the Most Potent PEP-Inhibiting Fractions 

Subfraction 9(6) of the hydrolysate E was the one that presented a PEP-inhibiting capacity, with an IC50 of 55 µg/mL. It consisted of 273 peptides that matched proteins in the database. Only 20 peptides, with a molecular mass ranging from 844 to 1541 Da, accounted for 81.5% of the total (Table 8). The presence of novel peptides was insignificant. The most abundant peptide was NWDDM(+15.99)EKIWHH, comprising nearly 37% of the total (Table 8). This peptide is a fragment of actin from the bivalve *Brevinucula verrillii* (accession number G3ET61_9BIVA, source Uniprot), indicating that it does not correspond to a collagen fragment. It was followed by the peptide SWFQQPK(+ 15.99)GL, with an abundance of 15.6%. This peptide is a fragment of the protein COL2A from *Sephia pharaonis* (accession number A0A812EDK2_SEPPH, source Uniprot). The sequence NWDDMEK, with hydroxylated methionine, was found in approximately 47% of the total peptides in the subfraction. This sequence was occasionally followed by EK, EKIW, EKIWH or EKIWHH. Although some peptides sharing a common sequence with these peptides have shown bioactive activity, none of them have been identified as PEP inhibitors. This is the case of the antioxidant peptide WDDMEK [59]. Moreover, seven of the most-abundant peptides contained leucine in the penultimate position (C2), representing about 14% of the total. Interestingly, tryptophan was present in the second position from the N-terminal in approximately 79% of the total peptides in the subfraction. It is worth noting that while ACE inhibitory peptides have been extensively studied, PEP-inhibiting peptides have not been widely identified. According to the BIOPEP-UWM database [54], the PEP-inhibiting peptides typically have a molecular weight of up to 2300 Da, with the most abundant ones having a molecular weight between 900 and 1100 Da. This is the range where the peptides were predominantly found in subfraction 9(6). Chanajon et al. [60] noted that longer peptides tend to be more effective inhibitors of PEP than shorter ones. Longer peptides are more likely to interact with multiple binding sites on the enzyme. PEP inhibitory peptides share a common structure, including the presence of proline at position C4 and hydrophobic and bulky amino acids at adjacent positions, as noted by Fülöp et al. [61] and Pripp [62]. Hydrophobic residues are important for PEP inhibition because they interact with residues with non-polar side chains in the S2 and S3 subsites [61,62], and bulky amino acids induce PEP inactivation. Most peptides found in subfraction 9(6) contain at least one Pro residue, in some cases in position C4 and adjacent to at least one hydrophobic residue (Table 8). Examples of such peptides include TMSPDFPELT, VIENPFLR and TRVPINF. The BIOPEP-UWM database [54] contains numerous peptides that inhibit PEP, some of which include proline residues in their sequence. However, it should be noted that proline is not always present in the position indicated by Fülöp et al. [61] and Pripp [62]. The presence of tryptophan in the second position in almost 80% of the peptides could also be related to the PEP inhibitory activity of the subfraction. Most PEP inhibitory peptides in the BIOPEP-UWM database [54] contain hydrophobic residues in this position, indicating their significance in peptide–enzyme interactions.

## 3. Materials and Methods

### 3.1. Enzyme and Chemicals

Angiotensin-converting enzyme I (ACE, EC 3.4.15.1), acetonitrile of HPLC grade and trifluoroacetic acid (TFA) were from Sigma Chemical Co. (St. Louis, MO, USA); Prolyl endopeptidase (PEP, EC 3.4.21.26) from Flavobacterium was purchased from Seikagaku Corp. (Tokyo, Japan). The chromogenic substrates (Z-Gly-Pro-7-amido-4-methylcoumarin) and Abz-GLY-PHe(NO2)-Pro were from Bachem Feinchemikalien (Bubendorf, Switzerland). Sigma Chemical Co. (St. Louis, MO, USA) provided porcine kidney Dipeptidyl Peptidase IV (DPP-IV, EC 3.4.14.5) and its chromogenic substrate (H-Gly-Pro-AMC-HBr), hippuryl-His-Leu, angiotensin II, vitamin B12 and aprotinin. Novozymes (Bagsværd, Denmark) kindly provided commercial proteases (Alcalase® 2.4 L, Esperase® 8.0 L, and Pancreatic Trypsin Novo 6.0S). All other analytical grade chemicals were purchased from Panreac Chemical Co. (Barcelona, Spain).

### 3.2. Raw Material 

The skins derived from the industrial processing of Argentine shortfin squid (*Illex argentinus*) were kindly provided by the company Antonio & Ricardo (Madrid, Spain). The skins were transported at a low temperature to the laboratory and immersed in cold water before being drained for an hour. Next, the skins were crushed in the presence of dry ice and treated with acetone (skin:acetone ratio of 1:3, w:v) for 2 h to remove pigments and lipids. After being rinsed twice with Milli-Q water (Millipore Co., New Bedford, MA, USA), the squid skin was stored at −20 °C until use.

### 3.3. Determination of the Proximate Composition of Squid Skin

The proximate composition of squid skin was determined by evaluating its moisture and ash contents according to AOAC standard protocols 930.15 and 942.05, respectively [63]. The protein content was determined using the Dumas method [64] and an LECO TruMac N analyser from Leco Corp. (AG Geleen, The Netherlands), with a conversion factor of 5.5. The total carbohydrates were determined using the phenol-sulfuric acid method [65].

### 3.4. Enzymatic Hydrolysis

#### 3.4.1. Measurement of Enzymatic Activity

The enzymatic activity of the enzymes used was measured using casein as a substrate, following the method described by Lajmi et al. [41]. One unit of protease activity was defined as the amount of enzyme required to release 1 µmol Tyr/min.

#### 3.4.2. Preparation of Protein Hydrolysates

Protein hydrolysis was conducted using Esperase, Alcalase or Trypsin, following the protocol of Bougatef et al. [66], at the pH and temperature conditions listed in Table 2. Three enzyme units were added per milligram of protein in the sample. The hydrolysis lasted for two hours while maintaining a constant pH, which was monitored using a pH-Stat Titromatic 1S (Crison Instruments, Barcelona, Spain). The enzymes were inactivated by heating at 80 °C for 15 min. The hydrolysates were then centrifuged at 15,200× *g* for 20 min at 4 °C using a Beckman Coulter J2-mc centrifuge (Brea, CA, USA). The supernatants were lyophilized and stored at −20 °C for future use.

### 3.5. Determination of the Degree of Hydrolysis

The degree of hydrolysis (DH) was determined using the method described by Adler-Nissen [67] and presented as a percentage of peptide bonds cleaved to the total number of peptide bonds found in the substrate.

### 3.6. Molecular Weight Analysis

Size exclusion chromatography (SEC) was utilized to obtain the molecular weight distribution of the hydrolysates, following the protocol of Martínez-Alvarez et al. [68]. SEC was carried out using an HPLC model LC-10ADVP (Shimadzu, Tokyo, Japan) and a Superdex Peptide PC 3.2/30 column (GE Healthcare, Barcelona, Spain) with a fractionation range between 7000–100 Da. The solvent utilized was 30% (*v*/*v*) acetonitrile containing 0.1% (*v*/*v*) TFA, at 25 °C, with a flow rate of 0.1 mL/min. Bovine serum albumin was used to determine the void volume, while aprotinin, angiotensin II, vitamin B12, N-hippuryl-His-leucine (HHL) and glycine were used as standards. Absorbance readings were taken at wavelengths of 214 and 280 nm.

### 3.7. Biological Activities

#### 3.7.1. ABTS Free-Radical-Scavenging Activity

ABTS (2,2′-azino-bis-[3-ethylbenzothiazoline]-6-sulfonic acid) radical-scavenging activity was determined according to Djellouli et al. [69]. The ABTS radical solution, which had been previously activated for 16 hour in darkness, was diluted in Milli-Q water to achieve an absorbance of approximately 0.70 ± 0.02 at a wavelength of 734 nm. Then, 20 μL of the sample (10 mg/mL) was combined with 980 μL of ABTS reagent and incubated in darkness at 30 °C for 10 min. Absorbance values were measured at 734 nm using a Shimadzu UV-1601 spectrophotometer (model CPS-240). Each sample was analysed in triplicate. The results were reported as milligram equivalents of ascorbic acid per gram of sample using an ascorbic acid standard curve.

#### 3.7.2. Ferric-Reducing Power Assay (FRAP)

The FRAP assay was performed according to Djellouli et al. [69]. In brief, 30 µL of sample (10 mg/mL) was incubated with 90 µL of Milli-Q water and 900 µL of FRAP reagent containing 10 mM of TPTZ and 20 mM of FeCl_3_ at 37 °C for 30 min. The absorbance was measured at 595 nm. Each sample was analysed in triplicate. The results were reported as mmol Fe^2+^ equivalents/g of sample and based on the FeSO_4_–7H_2_O standard curve.

#### 3.7.3. Angiotensin-I-Converting Enzyme Inhibitory Activity

The ACE inhibitory activity was measured using fluorescence spectrophotometry, following the method described by Bougatef et al. [66]. ACE was dissolved in 40 mL of 40% glycerol to achieve a final concentration of 25 mU/mL. Enzymatic activity was defined as the production of 1 μmol of hippuric acid from HHL/min in 50 mM of HEPES with 300 mM of NaCl at pH 8.3 and 37 °C. The assay was performed on a black 96-well microplate. The experiment included controls with 40 μL (1 mU) of enzyme and 160 μL of 150 mM Tris HCl buffer at pH 8.3 with 1.125 M of NaCl (working buffer). Blank samples with a working buffer and ACE inactivated with 5 M of HCl were also included. The ACE inhibitory activity of the samples (diluted in the working buffer) was tested at different concentrations, resulting in a final volume of 200 µL in each well. The microplate was incubated at 37 °C for 10 min before initiating the reaction with 100 μL of the fluorogenic substrate and 0.45 mM of Abz-Gly-Phe(NO2)-Pro, dissolved in the working buffer. Fluorescence was measured at excitation and emission wavelengths of 360 nm and 400 nm, respectively, for 15 min at 1-min intervals using a microplate reader. The study plotted data against time to determine the inhibitory activity of hydrolysates. The initial slopes were observed in the absence or presence of the test sample at various concentrations. The results were expressed as either the percentage of inhibition (if only one concentration of the sample was tested) or IC50 values.

#### 3.7.4. DPP-IV Inhibitory Activity

The DPP-IV inhibitory activity was evaluated using fluorometry in black 96-well microplates, following the method described by Sila et al. [40]. Briefly, 20 μL of DPP-IV (0.014 mg/mL; 10.64 mUnits), previously diluted in 100 mM Tris HCl buffer at pH 8, were incubated with 180 µL of working buffer (control samples), or with varying sample concentrations (final volume 200 μL per well) at 37 °C for 15 min. Prior to this, the samples were lyophilized and reconstituted in working buffer. The assay was initiated by adding 100 μL of assay buffer containing the fluorogenic substrate (H-Gly-Pro-AMC•HBr) at a final concentration of 25 μM. Fluorescence changes at 340 nm/440 nm were recorded at 1-min intervals for 15 min using a microplate reader. The inhibitory activity of hydrolysates was determined from the initial (maximal) slopes observed in the absence or presence of the test sample at various concentrations. The results were expressed as the percentage of inhibition or IC50 values.

#### 3.7.5. Prolyl Endopeptidase Inhibitory Activity

The protocol established by Lajmi et al. [41] was followed to determine PEP inhibitory activity in 96-well microplates. The samples were diluted to a concentration of 10 mg/mL in 0.1 M of sodium phosphate buffer at pH 7 (referred to as the working buffer). Briefly, 20 μL of enzyme (1 mU), defined as the enzyme activity producing 1 nmol of p-nitroaniline per minute at 30 °C and pH 7.0 from Z-Gly-Pro-pNA, was combined with either 180 μL of assay buffer (control sample) or different volumes of sample and assay buffer (final volume of 200 µl). Blanks were prepared with enzyme previously inactivated using 5 N of HCl. The mixture was then incubated at 30 °C for 15 min. The reaction began upon addition of 100 μL of 0.01 mM Z-GlyPro-AMC. Fluorescence was measured using a microplate reader at 1-min intervals for 20 min with excitation at 340 nm and emission at 440 nm. The inhibitory activity of hydrolysates was determined by comparing the initial slopes observed in the absence or presence of the test sample at various concentrations. Results were expressed as either the percentage of inhibition or IC50 values.

### 3.8. Fractionation of the Protein Hydrolysates

**Figure 1:**
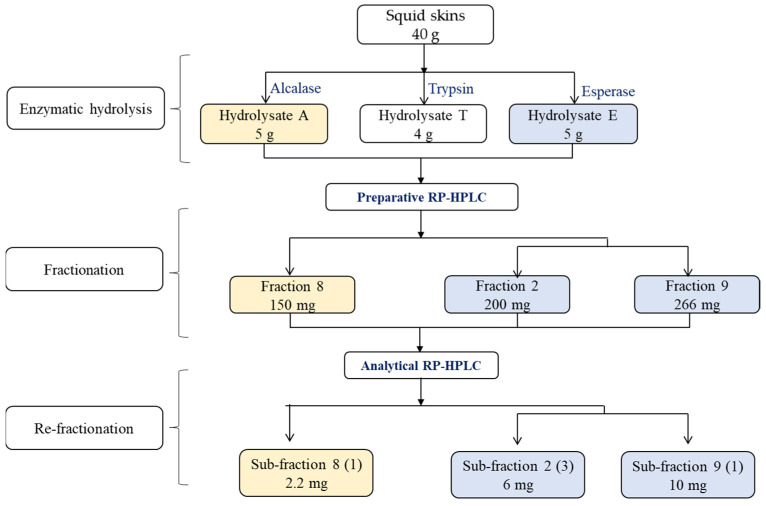
Procedure for purification and fractionation of bioactive fractions from squid skins.

The hydrolysates were freeze dried and dissolved in Milli-Q water (25 mg/mL), filtered through 0.45 µm (pore size) syringe filters and loaded onto a Europa protein C18 preparative column (particle size 5 µm, 25 × 1.0 cm, Teknokroma, Barcelona, Spain) coupled to a preparative HPLC (LC PREP 1260 Infinity Series, Agilent, Santa Clara, CA, USA). The solvents used were Milli-Q water (A) and acetonitrile (B), both containing 0.1% TFA. The following gradient of B was applied: 0–5% (0–10 min), 5–58% (10–30 min), 58–58% (30–35 min) and 58–0% (35–45 min), with a constant flow rate of 2.5 mL/min. Absorbance was measured at 215 nm (Figure 1).

The fractions that showed the highest ACE-, PEP- and DPP-IV-inhibiting ability were lyophilized, dissolved in Milli-Q water at a concentration of 100 mg/mL, and then filtered through syringe filters. The fractions were fractionated using RP-HPLC (Shimadzu LC-10ADVP) on a TRACER-Excel 120 ODS-A semi-preparative column (Teknokroma, Barcelona, Spain) with dimensions of 25 × 0.78 cm and a particle size of 5 μm. Milli-Q water + 0.1% TFA (A) and acetonitrile + 0.1% TFA (B) were used as solvents, with a flow rate that was set to 2 mL/min. Each individual sample was subjected to a distinct gradient. The fractions with the highest ACE-, DPP-IV- and PEP-inhibiting potency were selected for peptide composition analysis.

### 3.9. Peptide Identification

First, proteins in the raw material were identified to create a protein database for peptide identification. The skin sample was suspended in a mixture of 800 μL of 8 M urea and 100 μL of SDS, and then sonicated for 20 min. Following this, the mixture was centrifuged to remove any undissolved skin debris. An aliquot of 100 μL of supernatant was mixed with 400 μL of 50 mM TriEthylAmmoniumBicarbonate (TEAB) and digested with Trypsin-LysC on S-TrapTM microcolumns. The peptides present in the bioactive fractions and the digested skin protein extract underwent analysis using liquid nano chromatography (nano uEasy-nLC 1000, Thermo Scientific) coupled with a Q-Exactive HF high-resolution mass spectrometer (Thermo Scientific, Bremen, Germany). The peptides from the bioactive fractions (1 μg) were concentrated using an Acclaim PepMap 100 precolumn (Thermo Scientific, 20 mm × 75 μm ID, C18 with 3 μm particle diameter and 100 Å pore size) and further separated using an analytical C18 Picofrit reverse phase column (Thermo Scientific 500 mm × 75 μm ID, 2 μm C18 resin with 100 Å pore size) with an integrated spray tip. Separation was carried out with a flow rate of 250 nL/min and a gradient varying from 2% to 35% buffer B over a duration of 30 min (Buffer A, 0.1% formic acid in Milli-Q water). Buffer B, consisting of 0.1% formic acid in acetonitrile, was run for 10 min, followed by another 10-min run with B increasing to 40%. Peptides were detected using a Data Dependent Acquisition (DDA) method within an *m*/*z* mass range from 200–1200 Da. Up to 10 precursors with a charge between 2+ and 4+ were selected based on their intensity for fragmentation by High Collision Dissociation (HCD) in each MS scan. Peptides obtained from the protein extract were also scrutinized through the same process, but with a 150-min gradient and detected precursors with an *m*/*z* mass range from 300–1800.

Mascot 2.8 search engine (www.matrixscience.com access on 1 March 2024) was used to identify proteins and peptides from the skin extract using a database of Mollusca phylum sequences (573,737 sequences, https://www.uniprot.org access on 1 March 2024) and the database contaminants (247 sequences). The UP-squid database (12,257 sequences, 10 May 2023), obtained from the squid sequences in Uniprot, and the UP-collagen-Cephalo database (417 collagen sequences from cephalopods in Uniprot) were also used for this purpose. The probability filter applied for PSMs (Peptide Spectrum Matches), peptides and proteins identified with high confidence is a q value < 0.01 (high confidence). Values with q value < 0.05 corresponded to medium confidence and below this value they were considered low confidence. Correctly identified proteins were considered to be those that were below a false positive rate of 1% (FDR < 1%), and having at least 1 unique peptide identified with a Confidence Interval (CI) that exceeded 99%, i.e., the probability that the identified peptide was due to chance was less than 1% (q < 0.01). With the sequences of the total proteins identified in the squid skin sample, a database of 1742 sequences was obtained and used in the analysis of the subfractions from the MS/MS data using Peaks Studio v 10.5 software (Bioinformatic solution Inc., Waterloo, ON, Canada). Acceptance criteria for confidence peptides included a−10lgP ≥ 20, Unique Peptide ≥ 1 and PSM-FDR ≤ 1. Peptides whose sequences did not exceed the −10lgP scores established to be identified with confidence against the database were considered as de novo sequences. These can be peptides that are not in the database used, modified or with variants in their sequence. The de-novo-only peptides were classified and filtered according to a de novo ALC (Average Local Confidence) score, which is the average of the confidence of each of the amino acids in the de novo sequence. This ALC score was stablished as ≥70. The ‘Peaks PTM’ module was also used to find possible unspecified modifications (up to 313) with maximum up to 3 per peptide, both in peptides identified against the database and in those obtained by de novo sequencing as long as they exceed a certain quality (ALCscore > 15). 

## 4. Conclusions

Squid skin is a valuable byproduct of industrial processing due to its high protein content. Bioactive peptides can be obtained through controlled hydrolysis using Alcalase or Esperase. Both proteases are of microbial origin and can be obtained easily and at a relatively low cost compared to other proteins. Fractions enriched in potent ACE, DPP-IV or PEP inhibitory peptides may be useful as bioactive ingredients in human nutrition as nutraceuticals and food supplements. However, due to their long sequence, it will be necessary to protect them against gastrointestinal digestion. As well, further studies will be necessary to evaluate their ability to exert an in vivo effect. The use of the skins can help limit waste in squid processing, within the framework of a circular economy.

## Figures and Tables

**Table 1:** Composition of amino acid residues in the skin of the Argentine shortfin squid.

Types	Amino Acids	‰
Essential amino acid (EAA)	Leu (L)	55
	Arg (R)	53
	Thr (T)	42
	Lys (K)	42
	Val (V)	37
	Ile (I)	33
	Phe (F)	28
	Met (M)	22
	His (H)	14
Non-essential amino acids (NEAA)	Gly (G)	215
	Glx (E + Q)	98
	Asx (D + N)	87
	Ala (A)	84
	Ser (S)	55
	Pro (P)	42
	Tyr (Y)	18
	Cys (C)	6
	Hyp	58
	Hyl	12
THAA *		516
TEAA *		325
Pro + Hyp		100

* TEAA = total essential amino acid residues: Σ Ile + Leu + Lys + Met + Phe + Thr + Val + His + Arg. * THAA = total hydrophobic amino acid residues: Σ Pro + Ala + Val + Met + Gly + Ile + Leu + Phe.

**Table 2:** Proximate molecular weight of the most abundant peptides in the hydrolysates (in Daltons) and biological activities (antioxidant, PEP, DPP-IV and ACE) of squid skin hydrolysates obtained with different proteases. The IC50 values were expressed as mean ± SD (n = 3) in µg/mL. Different letters in the same column showed statistical differences among samples.

Enzyme	MW (Da)	ACE Inhibition (IC50)	PEP Inhibition (IC50)	DPP-IV Inhibition (IC50)	ABTS (mg Ascorbic acid/g)	FRAP (μM FeSO_4_/mg)
Esperase	720	53 ± 6 ^a^	38 ± 3 ^a^	673 ± 5 ^a^	16.61 ± 0.03 ^a^	41.62 ± 0.56 ^a^
Alcalase	1100	37 ± 3 ^b^	163 ± 21^b^	702 ± 3 ^b^	10.76 ± 0.03 ^b^	47.41 ± 2.77 ^b^
Trypsin	2500	74 ± 11 ^c^	129 ± 15 ^b^	863 ± 9 ^c^	18.68 ± 0.01 ^c^	41.95 ±1.30 ^a^

**Table 3:** PEP, DPP-IV and ACE inhibitory activity of the most active fractions obtained by chromatography (final concentration of sample in the well in parentheses).

Enzyme Used	Fraction	ACE (25 µg/mL)	DPP-IV (800 µg/mL)	PEP (400 µg/mL)
Esperase	2		84 ± 3%	
Esperase	9	66 ± 4%	86 ± 9%	55 ± 0%
Alcalase	8	52 ± 8%		59 ± 5%

**Table 4:** ACE, DPP-IV and PEP inhibitory activity of the most active subfractions, expressed as IC50 values in µg/mL. NA: no activity.

Enzyme Used	Fraction and Subfraction	ACE	DPP-IV	PEP
Esperase	2 (3)	NA	97 ± 6	NA
Esperase	9 (1)	16 ± 2	NA	NA
Esperase	9 (6)	NA	NA	55 ± 4
Alcalase	8 (1)	66 ± 10	NA	NA

**Table 5:** The most abundant peptides present in the subfraction 8(1) of the protein hydrolysate A. Average local confidence score (ALC). P(+15.99) = hydroxylation; K(+14.02) = methylation. Sub A = mutation (L by A); Sub V = mutation (M by V). M(+15.99) could correspond to an oxidised methionine or a phenylalanine.

Peptide	−10lgP	ALC Score	Mass (Da)	Error (ppm)	Abundance (%)
EGRGLP(+15.99)GL		87	813.433	−0.3	12.6
LGRGEP(+15.99)GL		84	813.433	−0.3	12.6
GEAGPP(+15.99)GLVGLP(+15.99)GER	96		1436.724	0.3	6.1
AGDDAPRAVFPS	77		1201.572	0.7	3.6
GTLGPDGPRGPSGL		94	1279.652	0.6	3.5
KIKIIAPPERKY	72		1454.897	0.7	2.5
TGPRTP(+15.99)GLAGPE		84	1167.587	0.4	2.2
AEREIVRDIKEKL	65		1597.915	−1.4	2.1
GATGP(+15.99)P(+15.99)GPIGHL	70		1104.554	1.3	2.1
EIK(+14.02)DIVTDVGM(+15.99)N	72		1362.670	2	1.7
NLLLEPH		93	834.459	−0.2	1.7
QGEQGIL(subA)GIP(+15.99)GPM(sub V)GPPGP(+15.99)KGH	53		2055.018	−7.5	1.6
NWDDM(+15.99)EKIWHH	82		1525.640	−0.7	1.6
LDVKLPN		95	797.464	−0.3	1.0
DIRPEVPEMVNQ	74		1425.692	1.4	1.0
LARKGVL		87	755.501	2.7	0.9
RAKLGVL		85	755.501	2.7	0.9
RVAPEEHPVL	77		1145.619	0.3	0.9
MYPGIADRM(+15.99)	61		1068.473	1.2	0.8
GDDAPRAVFPS	74		1130.535	1.1	0.8

**Table 6:** The most abundant peptides present in subfraction 9(1) from the hydrolysate prepared using Esperase. Average local confidence score (ALC). N(+.98)= deamidation; P(+15.99) and K(+15.99) = hydroxylation; H(+14.02) and K(+14.02) = methylation; Sub G = mutation (T by G); M(+15.99) could correspond to an oxidised methionine or a phenylalanine.

Peptide	−10lgP	ALC Score	Mass (Da)	Error (ppm)	Abundance (%)
SHPDLRLA		93	907.487	0	8.59
LGMP(+15.99)GRL		92	758.410	−0.4	8.48
GNK(+15.99)GPVGPL		88	853.464	1.6	8.29
KYPIEH(+14.02)GIVT	52.83		1169.644	0.8	7.40
NGK(+15.99)GPVGPL		90	853.464	1.6	4.15
HN(+.98)KIFIN	50.35		885.470	0.5	2.42
GERGPAGPVGVP(+15.99)GESGPP(+15.99)GPIGSR	84.89		2215.096	2.2	2.33
AVDGVP(+15.99)GNDGPVGSV		80	1354.635	2.1	1.89
LLLEPH		95	720.41	0.3	1.73
GTP(+15.99)GELGLP(+15.99)GELQK(+15.99)		83	1442.722	3	1.47
KVKDDWELR		90	1187.629	0.3	1.14
GHP(+15.99)GKDGLPT(sub G)FPGERGETG	43.58		1923.906	1.6	0.99
WDDM(+15.99)EKIWHH	56.47		1411.597	−0.1	0.81
LGPDGPRGPSGL		86	1121.582	1	0.81
LGPDGPRGAP(+15.99)GL		84	1121.582	1.6	0.81
KWDKVEKL		97	1044.596	1.6	0.80
TERGYSF	47.93		858.387	−0.4	0.79
M(+15.99)VPFPR		90	761.389	−0.4	0.77
KVGDLLR		98	799.491	−0.9	0.75
APRAVFPS		91	843.460	0.5	0.72

**Table 7:** The most abundant peptides in the DPP-IV-inhibiting subfraction 2(3) from the protein hydrolysate obtained using Esperase. N(+.98) and Q(+0.98) = deamidation; P(+15.99) and K(+15.99) = hydroxylation; Sub N = mutation (D by N0); P(+57–02)= carbamidomethyl.

Peptide	−10lgP	Mass (Da)	Error (ppm)	Abundance (%)
GEP(+15.99)GEQGPP(+15.99)GSP(+15.99)GPR	60.08	1465.641	6.7	21.62
K(+15.99)GPDGEHGRDGNSGSR	54.03	1640.724	1.9	16.85
GAP(+15.99)GPSGP(+15.99)P(+15.99)G	34.45	840.358	5.5	11.02
GNTGPAGP(+15.99)P(+15.99)GPQ	48.02	1080.482	6.3	10.62
P(+57.02)SGPP(+15.99)GQPGN	45.81	979.435	4.7	7.48
KGPDGEHGRDGNSGSR	47.62	1624.730	4.2	6.13
GADGERGPRGNRGDTGAS	40.86	1728.789	4.3	4.74
GPAGP(+15.99)P(+15.99)GAPG	42.73	808.369	4.7	4.43
GPTGP(+15.99)AGPAG	40.53	796.370	4.1	2.97
RHPEYAVSVLLR	52.09	1438.804	3.2	1.63
GAP(+15.99)GFP(+15.99)GPR	34.34	886.427	5.8	1.51
GPDGEHGRDGNSGSR	65.38	1496.635	2.4	1.51
IIAPPERKY	31.82	1085.623	3.8	1.30
GPP(+15.99)GN(+.98)PGP(+15.99)K	31.21	852.395	6.3	1.25
K(+15.99)GPDGEHGRDGD(sub N)SGSR	55.43	1641.708	8.7	0.99
GEHGRDGNSGSR	39.96	1227.534	2.1	0.94
SLLEGQDAK	33.12	959.492	4.2	0.75
GPDGDRGAP(+15.99)GPQ(+.98)G	37.07	1196.505	2.0	0.74

**Table 8:** The most abundant peptides present in the PEP-inhibiting subfraction 9(6) from the protein hydrolysate obtained using Esperase. N(+.98) = deamidation; P(+15.99) and K(+15.99) = hydroxylation; Sub G = mutation (T by G); H(+14.02) = methylation. M(+15.99) could correspond to an oxidised methionine or phenylalanine.

Peptide	−10lgP	ALC Score	Mass (Da)	Error (ppm)	Abundance (%)
SHPDLRLA		93	907.487	0	8.59
LGMP(+15.99)GRL		92	758.410	−0.4	8.48
GNK(+15.99)GPVGPL		88	853.464	1.6	8.29
KYPIEH(+14.02)GIVT	52.83		1169.644	0.8	7.40
NGK(+15.99)GPVGPL		90	853.464	1.6	4.15
HN(+.98)KIFIN	50.35		885.470	0.5	2.42
GERGPAGPVGVP(+15.99)GESGPP(+15.99)GPIGSR	84.89		2215.096	2.2	2.33
AVDGVP(+15.99)GNDGPVGSV		80	1354.635	2.1	1.89
LLLEPH		95	720.41	0.3	1.73
GTP(+15.99)GELGLP(+15.99)GELQK(+15.99)		83	1442.722	3	1.47
KVKDDWELR		90	1187.629	0.3	1.14
GHP(+15.99)GKDGLPT(sub G)FPGERGETG	43.58		1923.906	1.6	0.99
WDDM(+15.99)EKIWHH	56.47		1411.597	−0.1	0.81
LGPDGPRGPSGL		86	1121.582	1	0.81
LGPDGPRGAP(+15.99)GL		84	1121.582	1.6	0.81
KWDKVEKL		97	1044.596	1.6	0.80
TERGYSF	47.93		858.387	−0.4	0.79
M(+15.99)VPFPR		90	761.389	−0.4	0.77
KVGDLLR		98	799.491	−0.9	0.75
APRAVFPS		91	843.460	0.5	0.72

## Data Availability

Data are available upon request.
